# Incidences of obstetric outcomes and sample size calculations: A Danish national registry study based on all deliveries from 2008 to 2015

**DOI:** 10.1111/aogs.13700

**Published:** 2019-08-22

**Authors:** Stinne Hoegh, Line Thellesen, Karl Bang Christensen, Thomas Bergholt, Morten Hedegaard, Jette Led Sorensen

**Affiliations:** ^1^ Department of Obstetrics Juliane Marie Center for Children, Women and Reproduction, Rigshospitalet University of Copenhagen Copenhagen Denmark; ^2^ Section of Biostatistics Department of Public Health University of Copenhagen Copenhagen Denmark; ^3^ Hedegaard Clinic Copenhagen Denmark

**Keywords:** emergency cesarean section, incidence, methods, obstetric outcome, obstetrics, pregnancy outcome, research design, sample size

## Abstract

**Introduction:**

In high‐income countries the majority of pregnancies have a good outcome, and many adverse obstetric outcomes rarely occur. This makes demonstrating clinically relevant and statistically significant effects of new interventions a challenge. The objective of the study was to report incidences of important obstetric outcomes and to calculate sample sizes for tentative studies.

**Material and methods:**

The study was a registry‐based study. Data were retrieved from the Danish Medical Birth Registry and included all deliveries in Denmark from 2008 to 2015. The total population included 465 919 deliveries. The study population comprised intended vaginal deliveries with a single fetus in cephalic presentation at term (n = 381 567). Incidences were reported for 20 outcomes considering the relevance for the patients and the severity of the outcomes. We calculated the sample sizes required in tentative obstetric studies to detect risk reductions of 25 and 50%, for tests at the 5% level, using a power of 80 and 90%. For the randomized controlled trials we calculated the sample size required for comparing two proportions with equal‐sized groups. For the cohort study we calculated the sample size also required for two proportions but with unequal sized groups. Outcome measures for sample size calculation were neonatal mortality, Apgar score <7 at 5 minutes and emergency cesarean section.

**Results:**

The incidence of neonatal mortality, Apgar score <7 at 5 minutes and emergency cesarean section was 0.05, 0.58 and 10.5%, respectively. Using neonatal mortality as the outcome in a tentative randomized controlled trial with an expected risk reduction of 50% and power of 80%, our calculation showed a sample size of 195 036 deliveries. Using Apgar score <7 at 5 minutes or emergency cesarean section as the outcome, 16 254 and 818 deliveries, respectively, were required. In tentative cohort studies, the required sample sizes were larger due to the unequal proportion of exposed/non‐exposed women.

**Conclusions:**

Most adverse obstetric outcomes occur rarely; thus, very large sample sizes are required to achieve adequate statistical power in randomized controlled trials. Multicenter studies, international collaborations or alternative study designs to randomized controlled trials could be considered.

**Abbreviations**
ECS: emergency cesarean section
RCT: randomized controlled trial

**Key message**

The majority of obstetric outcomes occur with a very low incidence. Our sample size calculations showed that when using rare obstetric outcomes large sample sizes were required. Multicenter studies, international collaborations or alternative study designs to randomized controlled trials could be considered.

## 1 INTRODUCTION

In high‐income countries the majority of pregnancies have a good outcome, and many adverse obstetric outcomes rarely occur. This makes demonstrating clinically relevant and statistically significant effects of new interventions a challenge.

Randomized controlled trials (RCTs) are considered the gold standard for establishing causal inference in healthcare interventions, and are therefore frequently applied as study designs.^1^, ^2^ If RCTs have adequate statistical power, the expectation is that significant differences between groups will be a result of the intervention.^1^

To ensure the quality of scientific work, calculating and reporting a study's sample size is fundamental.^2^ However, sample size calculations are sparsely reported in scientific papers, and many trials do not achieve the target sample size stated before starting the trial.^3^ When a study is underpowered, there is a risk of not finding the true difference between the groups, affecting the quality of the study.^4^ Two obstetric papers from 1997‐2000 on the introduction of continuous electronic fetal monitoring in obstetric care and on the potential bias when comparing small and large maternity institutions when studying stillbirth rates, respectively, discuss the implications of study design, rare outcome measures and large sample sizes.^5^, ^6^ The two studies concluded that, for rare outcomes, very large sample sizes were needed to detect statistically significant differences between study groups. Even though these two studies emphasize the implications of sample size calculation in obstetric outcomes, many researchers still include rare outcomes in their study design without having sufficient power to do so.

Our study had three objectives: first, to report incidences of 16 obstetric outcomes; secondly, to calculate sample sizes for tentative studies using three selected outcomes: neonatal mortality, Apgar score <7 at 5 minutes and emergency cesarean section (ECS); and thirdly, to discuss the implications for study design in obstetrics when choosing outcome measures.

## 2 MATERIAL AND METHODS

### 2.1 Population and study design

The study was a registry‐based study. Data were retrieved from the Danish Medical Birth Registry and included all deliveries in Denmark from 2008 to 2015. The Danish Medical Birth Registry contains information on all deliveries in Denmark, thus providing data on the mother, the child, the pregnancy and the delivery.

The annual number of deliveries in Denmark is approximately 60 000, with 96‐98% of deliveries in public hospitals and 2‐3% at home, mostly attended by midwives from public maternity departments.^7^ Currently there are 23 public maternity departments in Denmark, all with access to specialists in obstetrics and anesthesiology. Midwives attend all deliveries and an obstetrician is only involved in the event of complications.

In this study we operated with two populations: the total population and the study population. We used the former, which included all deliveries in Denmark in the study period, to report incidences of important obstetric outcomes.

For calculating sample sizes for tentative studies, we used a study population that included all intended vaginal deliveries with a term (gestational age ≥37 weeks) singleton in cephalic presentation. Stillbirths and homebirths were excluded. Incidences of the obstetric outcomes were also reported for the study population.

### 2.2 Outcome measures

It is the scientific question raised that defines whether an obstetric event is an intervention, an outcome or even a population, not the event itself. In our study we have defined 16 relevant and used obstetric outcomes in the literature and are aware that these outcomes in other studies could be defined as interventions or constitute a study population.

We chose 16 obstetric outcomes for reporting incidences of the total population: preeclampsia, hemolysis, elevated liver enzymes, and low platelets (HELLP) syndrome, eclampsia, induction of labor, oxytocin augmentation, umbilical cord prolapse, shoulder dystocia, vacuum extraction, ECS, postpartum hemorrhage ≥1000 mL, manual exploration of the uterus, stillbirth, Apgar score <7 at 5 minutes, preterm delivery <37 weeks of gestation, low birthweight <2500 g and neonatal mortality. We chose these outcomes considering the relevance for the patients and the severity of the outcomes. For the study population we report incidences of 14 obstetric outcomes.

For sample size calculation in tentative studies, we chose three outcomes from the core outcome set for key stakeholders in maternity care: neonatal mortality, Apgar score <7 at 5 minutes and ECS.^8^ The chosen outcomes reflect different incidences: one extremely rare, one rare and one more common.

Neonatal mortality is defined as death before the age of 28 completed days after live birth.^9^ Apgar score is used to assess the condition of the newborn at 1 and 5 minutes after birth and it is a validated predictor of neonatal survival.^10^ The Apgar score at 5 minutes is the best predictor of neonatal survival.^10^ Cesarean section is linked to a wide range of complications, such as uterine rupture and abnormal invasive placenta, which leads to higher risk of maternal and neonatal morbidity and mortality.^11^

### 2.3 Statistical analyses

The data have been used as part of another study.^12^ Before analysis, the dataset was checked for logical errors. We recoded missing data for maternal weight and height with unrealistic values and checked whether there was consistency between the diagnosis of the delivery and the surgical intervention or procedure coded.

The selected outcomes are reported as incidences, both for the total population and for the study population. The incidences of neonatal mortality, Apgar score <7 at 5 minutes and ECS in the study population formed the basis for the tentative sample size calculations. We calculated the sample sizes for the comparison of two proportions, which necessitates a proportion of the outcome, and the researcher to consider the intervention effect and the desired maximum risk of statistical errors. The statistical tool is provided in Supporting Information Appendix S1.

We calculated the sample size necessary for tentative RCTs and cohort studies to be able to detect risk reductions of 25 and 50% at the 5% level with a power of 80 and 90%, respectively. For the RCTs we calculated the sample size required for comparing two proportions with equal‐sized groups (i.e. 1:1 ratio), whereas for the cohort study we calculated the sample size also required for two proportions but with unequal sized groups. We calculated sample sizes for proportion of exposed women of 5, 10 and 25%.^13^

Incidences were computed using IBM SPSS® version 24 (IBM Corp., Armonk, NY, USA) and sample size calculations were made using SAS® software package version 9.4 (SAS Institute, Cary, NC, USA). As missing data were rare, imputation was not applied.

### 2.4 Ethical approval

Approval was obtained from the Danish Data Protection Agency (file no.: 2012‐58‐0004). As this was a registry‐based study, ethical approval was not required according to the Danish Research Ethics Committee Law.^14^

## 3 RESULTS

From 2008 to 2015, there were 465 919 deliveries in Denmark. The study population, including intended vaginal deliveries with term singletons in cephalic presentation, consisted of 381 567 deliveries. There were missing data for Apgar score <7 at 5 minutes in 1260 deliveries (0.3%). There was no missing data for the variables neonatal mortality or ECS.

Table 1 shows the sociodemographic characteristics of the total population and the study population. In general, Danish women were most likely to deliver at term, to be 25‐34 years of age, to be non‐smokers, and to have a normal body mass index (i.e. 18.5‐24.9 kg/m^2^).

**Table 1:** Sociodemographic characteristics of the total population and the study population in Denmark from 2008 to 2015

Characteristics	Total population^a^	Study population^b^
	n (%)	n (%)
	465 919 (100)	381 567 (100)
Singleton deliveries	456 014 (97.9)	381 567 (100)
Twin deliveries	9794 (2.1)	—
Triplet/quadruplet deliveries	111 (0.0)	—
Breech deliveries	19 244 (4.1)	—
Singleton vaginal breech deliveries	1987 (0.5)	—
Planned cesarean section	43 407 (9.3)	—
Gestational age		
<37 weeks	30 544 (6.6)	—
37^+0^ to 39^+6^ weeks	206 986 (44.4)	159 973 (41.9)
≥40 weeks	228 193 (49.0)	221 594 (58.1)
Missing data	196 (0.0)	—
Maternal age (years)		
<25	57 907 (12.4)	49 985 (13.1)
25‐34	310 442 (66.6)	257 365 (67.4)
35‐39	81 335 (17.5)	62 532 (16.4)
≥40	16 235 (3.5)	11 685 (3.1)
Parity		
Nulliparous	212 445 (45.6)	177 674 (46.6)
Multiparous	248 976 (53.4)	203 893 (52.5)
Missing data	4498 (1.0)	3605 (0.9)
Smoking during pregnancy		
No	402 816 (86.5)	330 631 (86.7)
Smoking cessation during pregnancy	13 676 (2.9)	11 588 (3.0)
1‐20 cigarettes per day	41 061 (8.8)	33 215 (8.7)
>20 cigarettes per day	1417 (0.3)	1095 (0.3)
Missing data	6949 (1.5)	5038 (1.3)
Body mass index (kg/m^2^)		
<18.5	18 321 (3.9)	15 054 (3.9)
18.5‐24.9	276 318 (59.3)	229 585 (60.2)
25‐29.9	95 519 (20.5)	77 202 (20.2)
30‐34.9	37 128 (8.0)	29 342 (7.7)
≥35	20 302 (4.4)	15 618 (4.1)
Missing data	18 331 (3.9)	14 766 (3.9)

a The total population included all deliveries with gestational age 20^+0^ to 45^+0^.

b The study population included all term singleton (≥37 weeks of gestational age) with intended vaginal cephalic delivery.

Table 2 reports incidences of the 16 obstetric outcomes. Most outcomes occurred at a low incidence. The only outcomes with an incidence >10% were induction, oxytocin augmentation of labor and ECS. In the total population, the incidence of neonatal mortality, Apgar score <7 at 5 minutes and ECS was 0.4, 0.9 and 12.2%, respectively. In the study population, the incidence of neonatal mortality, Apgar score <7 at 5 minutes and ECS was 0.05% (95% confidence interval (CI) 0.04‐0.06), 0.58% (95% CI 0.55‐0.60) and 10.5% (95% CI; 10.4‐10.6), respectively.

**Table 2:** Incidences of obstetric outcomes in Denmark from 2008 to 2015

Outcome^a^	Total population^b^	Study population^c^
	n (%)	n (%)
	465 919 (100)	381 567 (100)
Pregnancy outcomes		
Preeclampsia	13 874 (3.0)	9836 (2.6)
HELLP	1177 (0.3)	472 (0.1)
Eclampsia	249 (0.05)	147 (0.04)
Induction of labor	102 499 (22.0)	93 174 (24.4)
Delivery outcomes		
Oxytocin augmentation	100 791 (21.6)	92 975 (24.4)
Umbilical cord prolapse	514 (0.1)	275 (0.1)
Shoulder dystocia	4449 (1.0)	4344 (1.1)
Vacuum extraction	32 816 (7.0)	30 943 (8.1)
Emergency cesarean section	56 619 (12.2)	40 416 (10.6)
Postpartum hemorrhage ≥1000 mL	10 627 (6.4)	8393 (6.2)
Manual exploration of the uterus	6634 (1.4)	4858 (1.3)
Neonatal outcomes		
Stillbirth	1721 (0.4)	—
Apgar score <7 at 5 minutes	4034 (0.9)	2201 (0.58)
Preterm delivery <37 weeks’ gestation	30 544 (6.6)	—
Low birthweight <2500 g	21 648 (4.6)	4949 (1.3)
Neonatal mortality	1310 (0.3)	184 (0.05)

Abbreviation: HELLP, hemolysis, elevated liver enzymes, and low platelets.

a One delivery can be represented more than once.

b The total population included all deliveries in Denmark from 2008 to 2015 with gestational age 20^+0^ to 45^+0^ weeks. In the event of multiple fetuses in one pregnancy, an outcome among one or more of the children counts.

c The study population included all term (≥37 weeks’ gestational age), singleton, intended vaginal cephalic delivery in Denmark from 2008 to 2015.

Table S1 reports incidences of the obstetric outcomes stratified by year.

**Figure 1:**
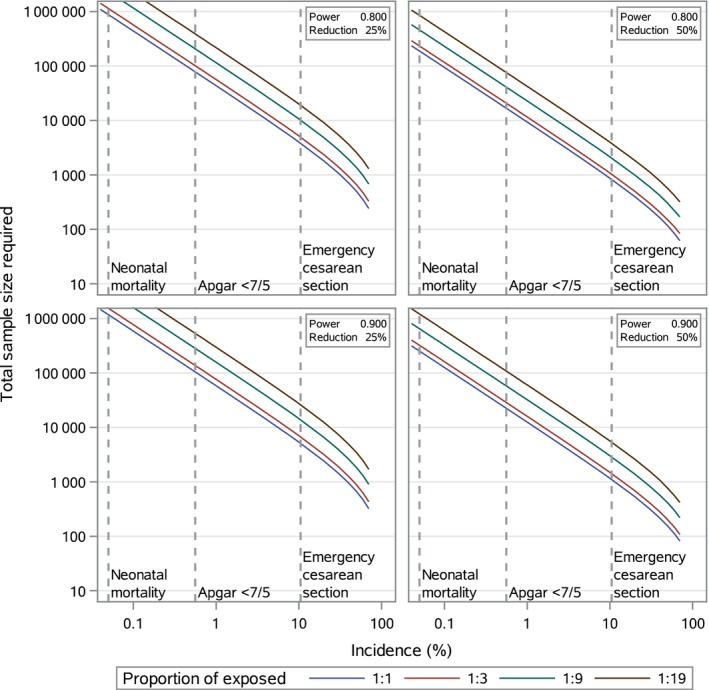
The total sample size required for tentative randomized controlled trials (1:1) and cohort studies (1:3, 1:9, 1:19) with neonatal mortality, Apgar score <7 at 5 minutes (<7/5) and emergency cesarean section as the outcome. Changes of a 25 and 50% reduction in outcomes are plotted against incidences, using an 80 and 90% power and a 5% significance level

Figure 1 and Table 3 report the sample sizes calculated for tentative RCTs and cohort studies. As shown, the incidence of the outcome measure affected the sample size. When using neonatal mortality with an incidence of 0.05% as the outcome in a tentative RCT with an expected risk reduction of 50% and power of 80%, our sample size calculation showed required sample size of 195 036 deliveries. Using Apgar score <7 at 5 minutes, with an incidence of 0.58%, as the outcome in a tentative RCT, with the same risk reduction and same power, 16 254 deliveries were required. For ECS with an incidence of 10.5%, 818 deliveries were required for a tentative RCT. Figure 1 and Table 3 also report the sample sizes required for studies with a power of 90% and for studies with a risk reduction of 25%.

**Table 3:** Sample sizes for tentative randomized controlled trials and cohort studies^a^

Outcome (incidence)								
Power	Risk reduction							
	50%				25%			
	Proportion of exposed				Proportion of exposed			
	5%	10%	25%	50%	5%	10%	25%	50%
Neonatal mortality (0.05%)								
80%	884 820	476 510	241 584	195 036	4 576 400	2 431 690	1 190 132	916 518
90%	1 259 520	673 250	334 172	261 096	6 280 940	3 327 940	1 615 172	1 226 960
Apgar score <7 at 5 minutes (0.58%)								
80%	73 680	39 680	20 112	16 254	377 260	200 460	98 112	75 786
90%	104 840	56 040	27 816	21 758	517 760	274 340	133 148	101 454
Emergency cesarean section (10.6%)								
80%	3740	2010	1016	818	18 820	10 000	4884	3764
90%	5300	2830	1400	1092	25 760	13 650	6620	5038

a Total sample sizes required for tentative classical randomized controlled trials with an allocation of 1:1 ratio (i.e. 50% exposed) and cohort studies with a proportion of exposed of 5, 10 and 25%.

Our results illustrate that an expected lower risk reduction increased the sample sizes. Using neonatal mortality as the outcome in a tentative RCT with 80% power and changing the risk reduction from 50% to 25% resulted in a fourfold increase in the required sample size to 916 518 deliveries. Using Apgar score <7 at 5 minutes or ECS as outcomes, the same fourfold increase in the required sample size was seen when the expected risk reduction changed from 50% to 25% (Figure 1, Table 3).

Our results furthermore illustrate that changing the power from 90% to 80% had a small impact on the required sample sizes. Using Apgar score <7 at 5 minutes as the outcome in a tentative RCT with 50% risk reduction and 80% power instead of 90%, the required sample size decreased from 21 758 to 16 254 deliveries. Using ECS as the outcome in an RCT with 50% risk reduction and 80% power instead of 90%, the required sample size decreased from 1092 to 818 deliveries (Figure 1, Table 3).

The study design furthermore affected the required sample sizes. When changing the proportion of exposed women from 50% to a smaller proportion, larger sample sizes were required. If Apgar score <7 at 5 minutes was used as the outcome in a tentative cohort study with 25% exposed instead of an RCT (50% exposed) with a power of 80% and risk reduction of 50%, the required sample size increased from 16 254 deliveries to 20 112 deliveries (Table 3). The same tendency was seen when the proportion of exposed women was even smaller. If the proportion of exposed was 10%, the required sample size was 39 680, and if the proportion of exposed was 5%, the sample size was further increased to 73 680 deliveries (Table 3).

Table S2 reports the required sample sizes for all 14 outcomes from the study population.

## 4 DISCUSSION

We found that the majority of obstetric outcomes occurred at a very low incidence.

Our sample size calculations showed that the choice of study design, the outcome incidence and the change from 50% to 25% in the risk reduction all contributed to the required sample size of the tentative studies. Changing the power from 90% to 80% did not have large impact on the required sample size.

A strength of our study is that the reported incidences are based on a large data source of 456 014 deliveries and the sample size calculations on a data source of 381 567 deliveries. The deliveries represent the period from 2008 to 2015, making the data fairly current.

Registry‐based research always involves the uncertainty associated with inaccurate reporting. Several studies show, however, that data from the Danish Medical Birth Register are valid in terms of diagnosis on most well‐defined outcomes, such as preeclampsia, birthweight, oxytocin augmentation of labor, vacuum extraction and cesarean section.^15^, ^16^

The study demonstrated that low incidence of the outcome affected the sample size. In Danish settings, if an RCT with 90% power was required to show a significant reduction of 25% of Apgar score <7 at 5 minutes, the study would take more than 2 years and require the inclusion of all deliveries. However, it might not be possible to include all eligible patients and some do not want to participate in the study. Thus, the time it takes to recruit patients will be longer than anticipated. This entails the researcher in the planning phase of an RCT to be realistic about recruitment and retention of participants in the study. This could be done through a feasibility study.

Our sample size calculations showed that a major contributor to the required sample size was the change from 50% to 25% in the risk reduction. Applying a risk reduction of 50% instead of 25% to the sample size calculation in a tentative RCT with a rare outcome such as Apgar score <7 at 5 minutes would still require a large‐scale multicenter study. Multicenter studies have the advantage of including more participants in shorter time. However, multicenter studies are considerably more complex to run than single‐site studies. Furthermore, the sample size calculations depend upon the assumption that the differences between the compared interventions in the centers are unbiased estimates of the same quantity. Based on previous studies, a reduction in risk of 50% or 25% in Apgar score <7 at 5 minutes is probably unrealistic.^17^, ^18^ A realistic and still clinically important reduction in Apgar score <7 at 5 minutes might be 10%, which would require an even larger sample size.

With a more common outcome such as ECS, conducting RCTs is more feasible because of the requirement of smaller sample sizes to achieve the adequate power. This might explain why ECS is often seen as an outcome in obstetric studies.^17^, ^19^, ^20^, ^21^ ECS may be a relevant outcome, but it is also easier to obtain power to show statistically significant results compared with a more rare outcome. Furthermore, in many studies an effect on the more common outcomes is often found and the interpretation is that a given intervention has only affected these common outcomes. The intervention, however, could potentially also have affected the rare outcomes, but the study might be underpowered to show this effect.

Meta‐analyses of RCTs are a way of increasing the power of the estimated intervention effect. However, meta‐analyses are, like single‐site studies, prone to risk of systematic and random error.^22^, ^23^

Sometimes used in studies with rare outcomes,^24^, ^25^, ^26^ composite outcomes combine several variables, which are considered to be equivalent, into one outcome to increase the total incidence of these outcomes. Composite outcomes enable the study to be performed with a smaller sample and/or in less time. However, composite outcomes often provide an unclear reflection of the effect because the outcomes are not necessarily equivalent in terms of severity or measurements, and it is possible that the exposure increases the risk of one complication and decreases the risk of another. In the latter situation, the possible effect of the exposure may be camouflaged.^27^

RCTs are considered the gold standard for establishing causality between exposure and outcome in healthcare interventions.^1^, ^2^ RCTs are usually expensive and time‐consuming, and not all research questions can be answered by interventional studies due to ethical considerations. A possible alternative study design to RCTs is observational studies, such as cohort or case–control studies. These designs facilitates studies using rare outcomes as they allow for inclusion of large populations due to no intervention and no treatment, just as the exposure does not have to be administered to the participants.^28^ Thus, even though our sample size calculations revealed larger sample sizes for cohort studies of unequal groups, also when studying rare outcomes such as Apgar score <7 at 5 minutes, obtaining the required sample size in a cohort study would be easier than in an RCT. This design would even allow studying rare outcomes of low exposure and also even with a relatively low reduction in risk. There is a limitation when studying extremely rare outcomes because the required sample sizes will be extremely large, making it impossible to recruit participants within a reasonable time frame. Cohort studies, however, have the limitations that they are prone to bias, the data may be inaccurate and misclassified, and deducing causal conclusions is not possible, weakening the study's internal validity. The International Network of Obstetric Survey Systems (INOSS), a multi‐country collaboration, facilitates studies of rare and severe outcomes in pregnancy and childbirth through international cooperation.^29^ International collaborative work on registry‐based data might be a good approach to obtain sufficient sample size when studying rare outcomes. Sometimes RCTs are not the most feasible study design, and many historical cases exist in which treatments with a convincing change are based on observational studies.^30^

The attention given to relevant outcome measures, as in the CROWN initiative^31^ is very important to obtain high quality evidence, but it is also essential to consider whether an RCT with rare outcomes is actually feasible, or if an alternative study design must be chosen. To our knowledge, the medical literature on sample size calculation and power regarding obstetric outcomes and choice of study design is sparse. Our results support the findings from two other studies investigating this issue. Mongelli et al demonstrated that when introducing electronic fetal monitoring, it was much easier to detect an increase in the incidences of cesarean section than a reduction in morbidity because of the different sample sizes and time needed to detect a significant change in the two outcomes.^5^ Moster et al demonstrated that large sample sizes were needed when comparisons of safety between different sizes of delivery units were made for low‐risk pregnancies, including stillbirth as the outcome measure.^6^ Our findings furthermore provide insights into sample sizes in relation to study design in both rare and more common obstetric outcomes.

## 5 CONCLUSION

Based on Danish national data from an 8‐year period, we found that several obstetric outcomes occur rarely. Consequently, very large sample sizes are required to achieve adequate statistical power in tentative RCTs. This necessity entails a risk of studies being underpowered or only showing an effect on common outcomes when an effect on rare outcomes might also exist.

Focusing on international multicenter collaboration and prioritizing a feasible study design can provide high quality evidence when investigating rare outcomes.

## CONFLICT OF INTEREST

The authors have stated explicitly that there are no conflicts of interest in connection with this article.
