# Supplementary material for: Incidences of obstetric outcomes and sample size calculations: A Danish national registry study based on all deliveries from 2008 to 2015
Source: Acta Obstet Gynecol Scand. 2019 Aug 22;99(1):34–41. doi: 10.1111/aogs.13700 (PMC6972555; doi:10.1111/aogs.13700)
Supplement: Supplementary file 1 [file AOGS-99-34-s001.docx]

**Appendix S1. Statistical tool used for sample size calculation**

Calculating sample sizes for the comparison of two proportions requires specification of the expected proportion of the outcomes $p_{1}$ and $p_{2}$ in the two groups (thereby indicating the expected intervention effect in the case of an intervetion study). Furthermore, the desired statistical power and the maximum tolerable risk of a statistical type I error must be specified. For a test of the null hypothesis

$$H_{0}:p_{1}=p_{2}$$

that two proportions $p_{1}=\frac{x_{1}}{n_{1}}$ and $p_{2}=\frac{x_{2}}{n_{2}}$ are equal the test statistic

$$z=\frac{p_{2}-p_{1}}{[{p(1-p)(\frac{1}{n_{1}}+\frac{1}{n_{2}})]}^{0.5}}$$

where $p={(x}_{1}+x_{2})/({n_{1}+n}_{2})$ is assumed to have a null distribution of N(0,1) and the power is

$$\Phi\left( \frac{\left( p_{2}-p_{1}-p_{0} \right)\left( Nw_{1}w_{2} \right)^{0.5}-z_{1-\frac{\alpha}{2}}{[{(w}_{1}p_{1}+w_{2}p_{2})(1-w_{1}p_{1}+w_{2}p_{2})]}^{0.5}}{{[{(w}_{2}p_{1}\left( 1-p_{1} \right)+w_{1}p_{2}(1-p_{2})]}^{0.5}} \right)$$

The required sample size is obtained by numerically inverting the power formula.

**SAS^®^ syntax**

**%let prop1=0.105921110;**

**%let prop2=0.052960555;**

**%let proc power;**

**twosamplefreq**

**groupproportions = (&prop1 &prop2)**

**ntotal = .**

**power = 0.80 0.90**

**alpha = .05;**

**run;**
