# Supplementary material for: Incidences of obstetric outcomes and sample size calculations: A Danish national registry study based on all deliveries from 2008 to 2015
Source: Acta Obstet Gynecol Scand. 2019 Aug 22;99(1):34–41. doi: 10.1111/aogs.13700 (PMC6972555; doi:10.1111/aogs.13700)
Supplement: Supplementary file 2 [file AOGS-99-34-s002.docx]

**Table S2**. Incidences of mode of delivery and obstetric outcomes in the total Danish population from 2008 to 2015 stratified by year.

| **Year** | **2008** | **2009** | **2010** | **2011** | **2012** | **2013** | **2014** | **2015** |
| --- | --- | --- | --- | --- | --- | --- | --- | --- |
| **All deliveries**^a^ | 63 274 | 61 133 | 61 653 | 57 301 | 56 208 | 54 448 | 55 653 | 56 249 |
| **N (% of all deliveries)** | | | | | | | | |
| **Singleton deliveries** | 61 820 (97.7) | 59 765 (97.8) | 60 309 (97.8) | 56 041 (97.8) | 54 967 (97.8) | 53 256 (97.8) | 54 573 (98.1) | 55 283 (98.3) |
| **Twin deliveries** | 1 437 (2.3) | 1 350 (2.2) | 1 335 (2.2) | 1 240 (2.2) | 1 230 (2.2) | 1 177 (2.2) | 1 070 (1.9) | 955 (1.7) |
| **Twin vaginal deliveries** | 618 (1.2) | 530 (1.1) | 529 (1.1) | 496 (1.1) | 461 (1.0) | 417 (1.0) | 415 (1.0) | 369 (0.8) |
| **Singleton breech deliveries** | 2 838 (4.5) | 2 683 (4.4) | 2 572 (4.2) | 2 456 (4.3) | 2 287 (4.1) | 2 121 (3.9) | 2 139 (3.8) | 2 148 (3.8) |
| **Singleton vaginal breech deliveries** | 268 (0.5) | 239 (0.5) | 236 (0.5) | 250 (0.6) | 250 (0.6) | 246 (0.6) | 252 (0.6) | 246 (0.6) |
| **Planned cesarean section** | 5 837 (9.2) | 5 808 (9.5) | 5 562 (9.0) | 5 402 (9.4) | 5 314 (9.5) | 5 216 (9.6) | 5 193 (9.3) | 5 075 (9.0) |
| **Preeclampsia** | 1 624 (2.5) | 1 767 (2.9) | 1 885 (3.1) | 1 873 (3.2) | 1 723 (3.0) | 1 718 (3.2) | 1 687 (3.0) | 1 597 (2.8) |
| **HELLP** | 163 (0.3) | 150 (0.2) | 162 (0.3) | 152 (0.3) | 140 (0.2) | 139 (0.3) | 137 (0.2) | 134 (0.2) |
| **Eclampsia** | 42 (0.07) | 27 (0.04) | 34 (0.06) | 26 (0.05) | 30 (0.05) | 30 (0.06) | 26 (0.05) | 34 (0.06) |
| **Induction of labor** | 11 035 (17.4) | 11 728 (19.2) | 12 605 (20.4) | 13 766 (24.0) | 14 432 (25.7) | 13 317 (24.5) | 12 763 (22.9) | 12 853 (22.9) |
| **Oxytocin augmentation** | 13 864 (21.9) | 13 665 (22.4) | 13 741 (22.3) | 13 242 (23.1) | 12 423 (22.1) | 10 864 (20.0) | 11 504 (20.7) | 11 488 (20.4) |
| **Umbilical cord prolapse** | 63 (0.1) | 56 (0.1) | 82 (0.1) | 69 (0.1) | 52 (0.1) | 79 (0.1) | 59 (0.1) | 54 (0.1) |
| **Shoulder dystocia** | 606 (1.0) | 605 (1.0) | 631 (1.0) | 604 (1.1) | 541 (1.0) | 500 (0.9) | 426 (0.8) | 536 (1.0) |
| **Vacuum extraction** | 4 962 (7.8) | 4 655 (7.6) | 4 439 (7.2) | 4 110 (7.2) | 3 773 (6.7) | 3 660 (6.7) | 3 678 (6.6) | 3 539 (6.3) |
| **Emergency cesarean section** | 7 651 (12.1) | 7 645 (12.5) | 7 703 (12.5) | 6 805 (11.9) | 6 601 (11.7) | 6 940 (12.7) | 6 752 (12.1) | 6 522 (11.6) |
| **Postpartum hemorrhage ≥1000 ml** | - | - | - | - | - | 3 550 (6.5) | 3 459 (6.2) | 3 618 (6.4) |
| **Manual exploration of the uterus** | 715 (1.1) | 701 (1.1) | 848 (1.4) | 880 (1.5) | 864 (1.5) | 895 (1.6) | 822 (1.5) | 909 (1.6) |
| **Stillbirth** | 257 (0.4) | 229 (0.4) | 230 (0.4) | 219 (0.4) | 209 (0.4) | 200 (0.4) | 205 (0.4) | 172 (0.3) |
| **Apgar score <7 at 5 minutes** | 507 (0.8) | 450 (0.7) | 541 (0.9) | 532 (0.9) | 526 (0.9) | 495 (0.9) | 494 (0.9) | 489 (0.9) |
| **Preterm delivery <37 weeks gestation** | 4 334 (6.8) | 4 079 (6.7) | 4 058 (6.6) | 3 793 (6.6) | 3 664 (6.5) | 3 457 (6.3) | 3 610 (6.5) | 3 549 (6.3) |
| **Low birthweight <2500 g** | 2 993 (4.7) | 2 836 (4.6) | 2 829 (4.6) | 2 747 (4.8) | 2 649 (4.7) | 2 497 (4.6) | 2 603 (4.7) | 2 494 (4.4) |
| **Neonatal mortality** | 190 (0.3) | 142 (0.2) | 164 (0.3) | 156 (0.3) | 169 (0.3) | 152 (0.3) | 169 (0.3) | 168 (0.3) |

HELLP; hemolysis, elevated liver enzymes, and low platelets. ^a^All deliveries in Denmark with gestational age 20+0 to 45+0. In the event of multiple fetuses in one pregnancy, an outcome among one or more of the children counts. One delivery can be represented more than once.
