# Supplementary material for: Incidences of obstetric outcomes and sample size calculations: A Danish national registry study based on all deliveries from 2008 to 2015
Source: Acta Obstet Gynecol Scand. 2019 Aug 22;99(1):34–41. doi: 10.1111/aogs.13700 (PMC6972555; doi:10.1111/aogs.13700)
Supplement: Supplementary file 3 [file AOGS-99-34-s003.docx]

| **Table S3.** Sample sizes required for tentative randomized controlled trials and cohort studies for all 14 outcomes in the study population^a^  **Outcomes (incidences)** | | | | | | | | | | |
| --- | --- | --- | --- | --- | --- | --- | --- | --- | --- | --- |
| **Power** | **Risk reduction** | | | | | | | | | |
|  | **50%** | | | | **25%** | | | | | |
|  | **Proportion of exposed** | | | | **Proportion of exposed** | | | | | |
|  | **5%** | **10%** | **25%** | **50%** | **5%** | **10%** | | **25%** | | **50%** |
| **Neonatal mortality (0.05%)** | | | | | | | | | | |
| **80%** | 884 820 | 476 510 | 241 584 | 195 036 | 4 576 400 | 2 431 690 | 1 190 132 | | 916 518 | |
| **90%** | 1 259 520 | 673 250 | 334 172 | 261 096 | 6 280 940 | 3 327 940 | 1 615 172 | | 1 226 960 | |
| **Apgar score <7 at 5 minutes (0.58%)** | | | | | | | | | | |
| **80%** | 73 680 | 39 680 | 20 112 | 16 254 | 377 260 | 200 460 | 98 112 | | 75 786 | |
| **90%** | 104 840 | 56 040 | 27 816 | 21 758 | 517 760 | 274 340 | 133 148 | | 101 454 | |
| **Emergency cesarean section (10.6%)** | | | | | | | | | | |
| **80%** | 3740 | 2010 | 1016 | 818 | 18 820 | 10 000 | 4884 | | 3764 | |
| **90%** | 5300 | 2830 | 1400 | 1092 | 25 760 | 13 650 | 6620 | | 5038 | |
| **Preeclampsia (2.6%)** | | | | | | | | | | |
| **80%** | 16 260 | 8760 | 4436 | 3582 | 82 980 | 44 090 | 21 572 | | 16 662 | |
| **90%** | 23 120 | 12 360 | 6132 | 4794 | 113 840 | 60 310 | 29 268 | | 22 306 | |
| **HELLP (0.1%)** | | | | | | | | | | |
| **80%** | 345 320 | 185 960 | 94 268 | 76 212 | 1 768 920 | 939 950 | 460 072 | | 355 546 | |
| **90%** | 491 500 | 262 710 | 130 392 | 102 024 | 2 427 980 | 1 286 480 | 624 404 | | 475 974 | |
| **Eclampsia (0.04%)** | | | | | | | | | | |
| **80%** | 1 115 280 | 600 560 | 304 400 | 245 516 | 5 710 160 | 3 034 160 | 1 485 064 | | 1 142 048 | |
| **90%** | 1 587 220 | 848 360 | 421 016 | 328 676 | 7 837 360 | 4 152 660 | 2 015 484 | | 1 528 878 | |
| **Induction of labor (24.4%)** | | | | | | | | | | |
| **80%** | 1460 | 780 | 396 | 314 | 7140 | 3780 | 1844 | | 1414 | |
| **90%** | 2040 | 1090 | 540 | 418 | 9720 | 5150 | 2492 | | 1892 | |
| **Oxytocin augmentation (24.4%)** | | | | | | | | | | |
| **80%** | 1460 | 790 | 396 | 314 | 7160 | 3790 | 1848 | | 1418 | |
| **90%** | 2040 | 1090 | 540 | 420 | 9740 | 5160 | 2500 | | 1896 | |
| **Umbilical cord prolapse (0.1%)** | | | | | | | | | | |
| **80%** | 589 300 | 317 380 | 160 928 | 130 312 | 3 040 100 | 1 615 400 | 790 676 | | 612 870 | |
| **90%** | 838 960 | 448 470 | 222 616 | 174 450 | 4 172 740 | 2 210 950 | 1 073 096 | | 820 458 | |
| **Shoulder dystocia (1.1%)** | | | | | | | | | | |
| **80%** | 37 180 | 20 020 | 10 148 | 8200 | 190 120 | 101 020 | 49 440 | | 38 208 | |
| **90%** | 52 880 | 28 270 | 14 028 | 10 976 | 260 900 | 138 240 | 67 088 | | 51 148 | |
| **Vacuum extraction (8.1%)** | | | | | | | | | | |
| **80%** | 4980 | 2680 | 1352 | 1090 | 25 140 | 13 350 | 6528 | | 5034 | |
| **90%** | 7040 | 3770 | 1868 | 1458 | 34 440 | 18 240 | 8848 | | 6738 | |
| **Postpartum hemorrhage ≥ 1000 ml (6.2%)** | | | | | | | | | | |
| **80%** | 19 100 | 10 290 | 5212 | 4210 | 97 580 | 51 850 | 25 372 | | 19 596 | |
| **90%** | 27 160 | 14 520 | 7204 | 5634 | 133 880 | 70 930 | 34 424 | | 26 232 | |
| **Manual exploration of the uterus (1.3%)** | | | | | | | | | | |
| **80%** | 33 220 | 17 890 | 9068 | 7326 | 169 920 | 90 280 | 44 184 | | 34 142 | |
| **90%** | 47 260 | 25 260 | 12 536 | 9806 | 233 160 | 123 540 | 59 956 | | 45 706 | |
| **Low birth weight <2500 g (1.3%)** | | | | | | | | | | |
| **80%** | 32 600 | 17 560 | 8900 | 7190 | 166 820 | 88 640 | 43 376 | | 33 510 | |
| **90%** | 46 380 | 24 790 | 12 304 | 9624 | 228 900 | 121 290 | 58 864 | | 44 860 | |

HELLP; hemolysis, elevated liver enzymes, and low platelets. ^a^ Total sample sizes required for tentative classical randomized controlled trials with an allocation of 1:1 ratio (i.e. 50% exposed) and cohort studies with a proportion of exposed of 5%, 10% and 25%.
